# Web-Based Explainable Machine Learning-Based Drug Surveillance for Predicting Sunitinib- and Sorafenib-Associated Thyroid Dysfunction: Model Development and Validation Study

**DOI:** 10.2196/67767

**Published:** 2025-04-10

**Authors:** Fan-Ying Chan, Yi-En Ku, Wen-Nung Lie, Hsiang-Yin Chen

**Affiliations:** 1 Department of Clinical Pharmacy, College of Pharmacy, Taipei Medical University, 250 Wuxing St, Xinyi Dist, Taipei, 11031, Taiwan, 886 2-2736-1661; 2 Department of Electrical Engineering, National Chung Cheng University, Chiayi, Taiwan; 3 Department of Pharmacy, Wan Fang Hospital, Taipei Medical University, Taipei, Taiwan

**Keywords:** thyroid dysfunction, machine learning, cancer, sunitinib, sorafenib, TKI, tyrosine kinase inhibitor

## Abstract

**Background:**

Unlike one-snap data collection methods that only identify high-risk patients, machine learning models using time-series data can predict adverse events and aid in the timely management of cancer.

**Objective:**

This study aimed to develop and validate machine learning models for sunitinib- and sorafenib-associated thyroid dysfunction using a time-series data collection approach.

**Methods:**

Time series data of patients first prescribed sunitinib or sorafenib were collected from a deidentified clinical research database. Logistic regression, random forest, adaptive Boosting, Light Gradient-Boosting Machine, and Gradient Boosting Decision Tree were used to develop the models. Prediction performances were compared using the accuracy, precision, recall, F1-score, area under the receiver operating characteristic curve, and area under the precision-recall curve. The optimal threshold for the best-performing model was selected based on the maximum F1-score. SHapley Additive exPlanations analysis was conducted to assess feature importance and contributions at both the cohort and patient levels.

**Results:**

The training cohort included 609 patients, while the temporal validation cohort had 198 patients. The Gradient Boosting Decision Tree model without resampling outperformed other models, with area under the precision-recall curve of 0.600, area under the receiver operating characteristic curve of 0.876, and F1-score of 0.583 after adjusting the threshold. The SHapley Additive exPlanations analysis identified higher cholesterol levels, longer summed days of medication use, and clear cell adenocarcinoma histology as the most important features. The final model was further integrated into a web-based application.

**Conclusions:**

This model can serve as an explainable adverse drug reaction surveillance system for predicting sunitinib- and sorafenib-associated thyroid dysfunction.

## Introduction

### Background

Sunitinib- and sorafenib-associated thyroid dysfunction are time-varying and underreported adverse drug reactions (ADR). Despite the efficacy of multitargeted tyrosine kinase inhibitors TKI as first- or second-line therapies for solid and hematologic cancers, thyroid dysfunction—especially hypothyroidism—may complicate treatment regimens using sunitinib and sorafenib. Although not life-threatening, such adverse events can lead to a suboptimal quality of life, physical discomfort, or the need for thyroid dysfunction treatment [1,2]. Incidences of thyroid dysfunction vary from 10%- to 85% for sunitinib and from 6.3% to 27% for sorafenib [3-6]. Hypothyroidism develops late and is prolonged, with onset ranging from 5 to 20 months [1,6-8]. Due to its high variability and difficulty in predicting, close monitoring and proactive ADR surveillance with machine learning (ML) models may be warranted to manage sunitinib- and sorafenib-associated thyroid dysfunction.

Incorporating real-time laboratory data can enhance the performance of ML models in predicting thyroid adverse events. Previous studies have demonstrated the effectiveness of ML in predicting thyroid dysfunction, such as amiodarone-induced dysfunction using time-series data collection methods with a robust performance [9]. In contrast, predicting immune checkpoint inhibitor-induced adverse thyroid events without continuous laboratory testing resulted in an area under the precision-recall curve (AUPRC) of 0.510 [10]. Another study developed a predictive model for immune checkpoint inhibitor-induced adverse thyroid events without detailed time-point data collection, achieving an area under the receiver operating characteristic curve (AUROC) of 0.885 with thyroid-related features [11]. However, no studies have focused on predicting thyroid adverse events caused by TKIs such as sunitinib and sorafenib. Adopting timely data collection with detailed clinical biochemical tests to develop thyroid dysfunction surveillance systems can help clinicians timely adjust TKI treatment by balancing risks and benefits.

Applying model interpretation and web-based applications can alleviate the black-box drawback of ML models and enhance understanding of ADRs. The SHapley Additive exPlanations (SHAP) analysis is a mathematical method that has become increasingly popular for explaining ML models [12-14]. It is based on game theory concepts that calculate feature importance and contributions to predicted outcomes at both population and individual levels [15]. Model deployment in web-based or smartphone applications were constructed in recent years to strengthen the scalability of predicting specific diseases or ADRs [13,16,17]. Through a user-friendly interface and explainable model, applications can provide clinicians with a greater understanding and actionable preventive remedies for their patients. These techniques successfully have increased the interpretability and accessibility of ML models to support clinical decision-making.

The landscape of drug surveillance has experienced notable changes, shifting from passive reporting systems or rules-based alerting systems to active identification of adverse drug events, driven by advancements in electronic health records and artificial intelligence. Traditional methods often rely on spontaneous reporting or rules-based alerting, which may lead to underreporting and delays in identifying ADRs. The ML models, particularly tree-based algorithms, can analyze large-scale clinical data in real time, proactively detecting ADRs and enhancing medication safety at the point of care [18]. By continuously collecting and analyzing patient data, ML-driven drug surveillance based on time-series data extraction enables dynamic risk assessment, facilitating early intervention and personalized treatment adjustments. Given the underreported and time-varying nature of thyroid dysfunction, this progressive approach can serve as a valuable tool for predicting ADRs in patients undergoing TKI therapy.

### Objective

The objective of this study was to develop and validate progressive ML predictive models for sunitinib- and sorafenib-induced thyroid dysfunction. Five algorithms—logistic regression (LR), random forest (RF), Gradient Boosting Decision Tree (GBDT), Light Gradient Boosting Machine (LGBM), and Adaptive Boosting (AdaBoost)—were used to construct the models. The specific aims of this study were (1) to use time-series data collected at the baseline, and at 1, 2, 3, 4, 5, 6, 9, 12, 18, 24, 30, and 36 months after the index date to generate predictive models using the five algorithms; (2) to select the best model by comparing the accuracy, precision, recall, F1-score, AUROC, and AUPRC, after adjusting for the optimal threshold; (3) to interpret the best-performing model with a SHAP analysis to analyze feature importance levels and contributions, comparing findings with a previous amiodarone-induced thyroid dysfunction predictive model; and (4) to deploy the best-performing model by constructing a web-based application.

## Methods

### Ethical Considerations

This retrospective study used the deidentified Clinical Research Database (CRD) which includes data from Taipei Medical University Hospital, Wan Fang Hospital, and Shuang Ho Hospital of the TMU health care system. Ethical review for this study was waived by the TMU-Joint Institutional Review Board (approval no.: N202202053). As the data had been deidentified, the requirement for informed consent was waived. No compensation was provided to the participants.

### Study Design and Patient Cohort

**Figure 1.**
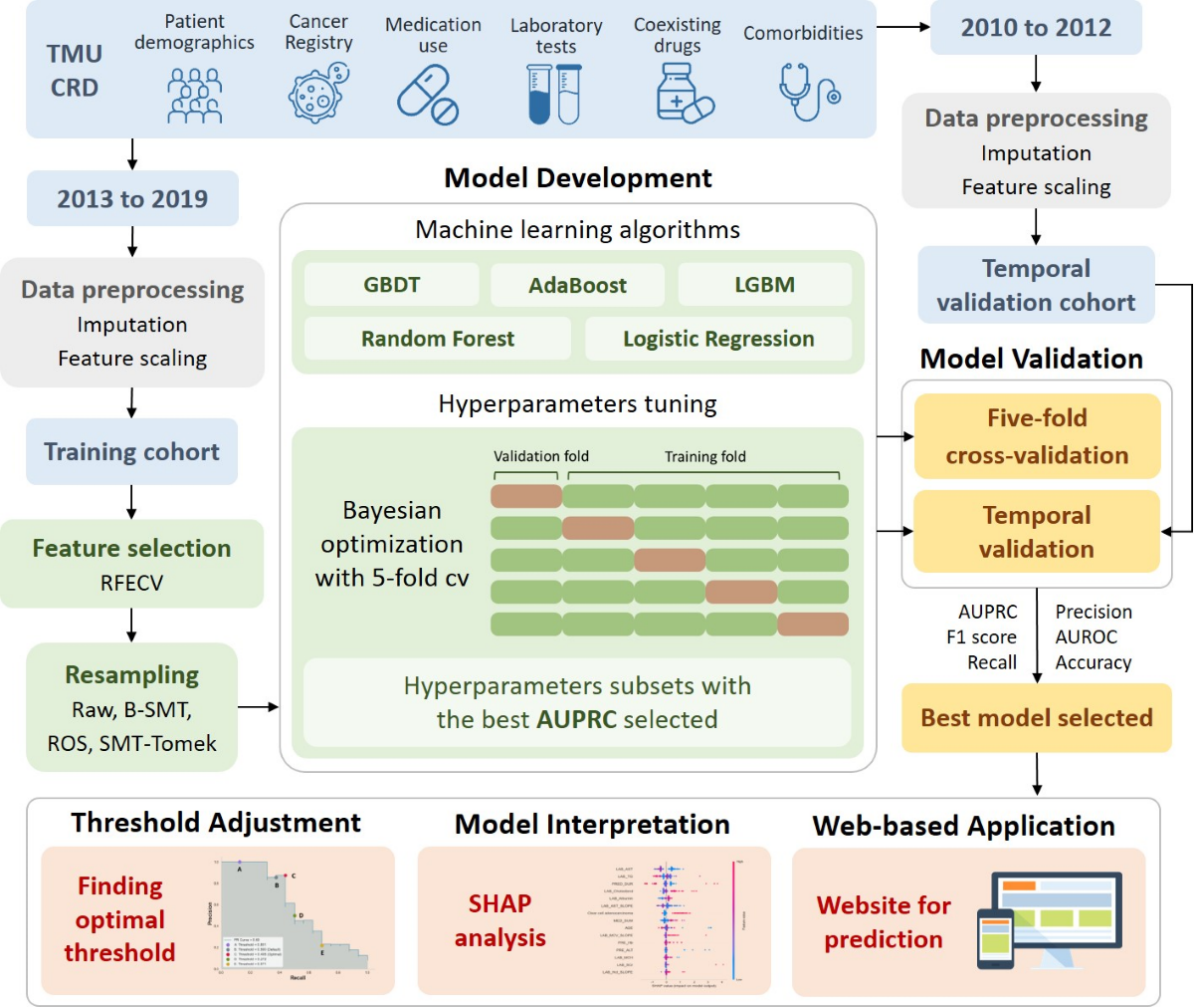
Study design of the proposed models. The study process included data collection, data splitting, data preprocessing, feature selection, resampling, model development, model validation, threshold adjustment, model interpretation, and development of a web-based application. AdaBoost: Adaptive Boosting; AUPRC: area under the precision recall curve; AUROC: area under the receiver operating characteristic curve; B-SMT: Oversampling with the Borderline Synthetic Minority Oversampling Technique; CRD: clinical research database; GBDT: Gradient Boosting Decision Tree; LGBM: Light Gradient Boosting Machine; RFECV: Recursive Feature Elimination with Cross-Validation; SMT: Synthetic Minority Oversampling Technique; TMU: Taipei Medical University.

Data from patients who were first prescribed sunitinib or sorafenib from 2013 to 2019 were collected as the derivation (training) cohort, whereas data from patients treated between 2010 and 2012 were collected as a temporal validation (testing) cohort. The data-splitting time point was chosen considering a common proportion of 7:3 for the training and testing cohorts and similar incidences of thyroid dysfunction in both cohorts for better model development and validation. More recent data were used for model training to better reflect current clinical practice, allowing models to be developed with up-to-date treatment patterns and patient characteristics. Patients were excluded if they were younger than 18 years, pregnant, with a history of hypothyroidism, had a diagnosis of thyroid carcinoma, had undergone thyroidectomy, used levothyroxine, or had abnormal thyroid-stimulating hormone (TSH) levels within 1 year before the index date, which was the first day of sunitinib or sorafenib use. Patients were also excluded if they were lost to follow up. Each individual was followed up for 3 years, and data were collected until the end of the study period, loss to follow-up, death, or occurrence of thyroid dysfunction. Figure 1 demonstrates the study design of the proposed models. This study followed the Transparent Reporting of a multivariable prediction model for Individual Prognosis Or Diagnosis + Artificial Intelligence (TRIPOD +  AI) reporting guideline [19]. Registration was not required, and no separate study protocol was prepared.

### Time-Series Data Collection and Preprocessing

Variables including patient demographics, cancer-related information, medication use, laboratory tests, coexisting drugs, and comorbidities were collected from the CRD. Patient demographics including age, gender, and cancer-related information were collected once at the baseline. Laboratory tests, medication use, comorbidities, coexisting drugs, weight, and body mass index (BMI) were collected at the baseline, and at 1, 2, 3, 4, 5, 6, 9, 12, 18, 24, 30, and 36 months after the index date (Multimedia Appendix 1). Reengineering features included calculating the cumulative dose, summed days of medication, duration of sunitinib or sorafenib, and the slope of laboratory tests at recent and previous data collection time points. The number of coexisting thyroid-related drugs was calculated to determine whether the aggregated use of these medications increased the risk of thyroid dysfunction.

Data preprocessing including missing value imputation and feature scaling was performed to ensure data quality. Features with over 80% missing values, except for the TSH level, were first excluded from the model. Missing values were imputed using multivariate imputation by chained equations [20]. If previous data were traceable, missing values, using time-series data collection, were precisely imputed using the last observation carried forward. Missing values in laboratory test slopes were imputed with zero. A feature-scaling method—the robust scaler—was used to reduce the effect of extreme values of continuous variables. Features were first selected through a literature review and clinical domain knowledge to identify thyroid-related variables. Recursive feature elimination, a feature selection technique that recursively removes the least important features with 5-fold cross-validation was then used, and the AUROC was considered to select optimal features for each model. The codebook and missing rates of each variable in the training and testing set are described in Multimedia Appendix 2.

### Prediction Outcome

The predictive outcome of this study was the occurrence of sunitinib- or sorafenib-associated thyroid dysfunction. Patients were defined as a TSH level ≥4 mIU/L, a diagnosis of hypothyroidism with International Classification of Diseases codes (*ICD-9-CM*; *243* or *244* or *ICD-10*; *E02* or *E03)*, or use of levothyroxine (Anatomical Therapeutic Chemical code: H03AA02). These criteria were defined following previous studies that focused on drug-induced thyroid dysfunction [9,21,22].

### Model Development and Validation

The study used three resampling strategies and raw data applied to the five algorithms to develop 20 machine learning models. The five algorithms included LR, RF, AdaBoost [23], LGBM [24], and GBDT [25]. Resampling strategies used were random over-sampling (ROS), borderline synthetic minority oversampling technique (B-SMT), and a combination of over- and under-sampling using SMT and Tomek links (STMK). Bayesian optimization with 5-fold cross-validation and the AUPRC were considered for hyperparameter tuning [26]. For boosting models, hyperparameters such as learning_rate and n_estimators were optimized to balance convergence speed and model complexity. Specifically, in LGBM, subsample and colsample_bytree were fine-tuned to mitigate overfitting. Key parameters including max_depth, min_samples_split, and min_samples_leaf were adjusted to improve generalization and prevent overfitting for RF. Table S3.1 in Multimedia Appendix 3 lists the ranges of hyperparameters used for tuning in each algorithm. Pseudocodes of the model development process are listed in Multimedia Appendix 4.

A stratified 5-fold cross-validation was first used for internal validation of the training dataset, and the performances of the 20 models were evaluated using the temporal validation cohort. The accuracy, precision, recall, F1-score, AUROC, and AUPRC were the metrics used for evaluating model performance. Model performance was compared and evaluated following the sequence of the highest AUPRC, AUROC value, and F1-score to select the best-performing model.

### Threshold Adjustment, Model Interpretation, and Web-Based Application

To optimize the performance of the best-performing model, different thresholds were selected on the PRC based on different percentages of outcome predictions. Five cutoff points were chosen: (1) a high-risk threshold for identifying a lower number of patients potentially at risk; (2) a default threshold of 0.50; (3) an optimal threshold based on the maximum F1-score; (4) an equal threshold where precision, recall, and F1-score were equal, and (5) the low-risk threshold for identifying a higher number of patients potentially at risk. The F1-score, recall, precision, and accuracy were then compared for these 5 thresholds. The best model was further examined by SHAP analysis to explain feature importance and contribution at both the population and individual levels [27]. At the population level, SHAP summary plots were used to demonstrate feature importance and how the top-ranked features impacted outcome predictions. On the other hand, SHAP force plots were used to visualize how the features contributed to outcome predictions in specific patients.

The model was further integrated into a web-based application to increase the accessibility of the model. The Python Flask framework was used to develop the application programming interface. The value of each feature served as input data that were fed back into the model to generate a predictive probability of thyroid dysfunction. The consistency of feature scaling between input data and training data was ensured by applying the same scaling transformation. The input variables with missing values were imputed with the last observation or the same multivariate imputation by chained equations transformation applied during model development to ensure consistency in predictions.

### Statistical Analysis

Patient characteristics were evaluated with independent *t* tests or the Wilcoxon rank-sum test for continuous variables, and the *χ*^2^ test or Fisher exact test for categorical variables. A two-sided *P* value <.05 was considered statistically significant. Data were analyzed using SAS (version 9.4; SAS Institute), Python (version 3.9.5; Python Software Foundation), and R software (version 4.2.2; R Foundation for Statistical Computing). The statistical significance of the AUPRC was calculated using MedCalc software (version 22.001).

## Results

### Patient Characteristics and Multivariate Analysis

This study enlisted data from 900 patients prescribed sunitinib or sorafenib from initial screening from the CRD. A STROBE (Strengthening The Reporting of Observational Studies in Epidemiology) flowchart of patient selection is described in Multimedia Appendix 5. After applying the exclusion criteria, 807 patients remained. Patient characteristics and multivariate analysis of the training and temporal validation cohorts are listed in Multimedia Appendix 6. The training cohort contained 609 patients, with 52 (8.5%) patients developing thyroid dysfunction, while 16 (8.1%) patients experienced thyroid dysfunction in the temporal validation cohort of 198 patients. There were no significant differences in age or gender in the derivation or temporal validation cohort. Cancer type, histology, aspartate aminotransferase (AST), and bilirubin levels significantly differed (*P*<.001) between patients with and those without thyroid dysfunction in the two cohorts.

### Model Development and Validation

The total number of features selected by recursive feature elimination were 20, 18, 15, 40, and 20 in GBDT, AdaBoost, LGBM, RF, and LR, respectively. The most frequently selected features were recent laboratory tests including AST, alanine transaminase (ALT), bilirubin, cholesterol, and triglyceride levels. Patient demographics such as age and BMI summed days of medication use, and follow-up duration were also among the top-ranked selected features (Multimedia Appendix 7). The optimal hyperparameter subsets selected by Bayesian optimization are listed in Table S3.2 in Multimedia Appendix 3.

**Figure 2.**
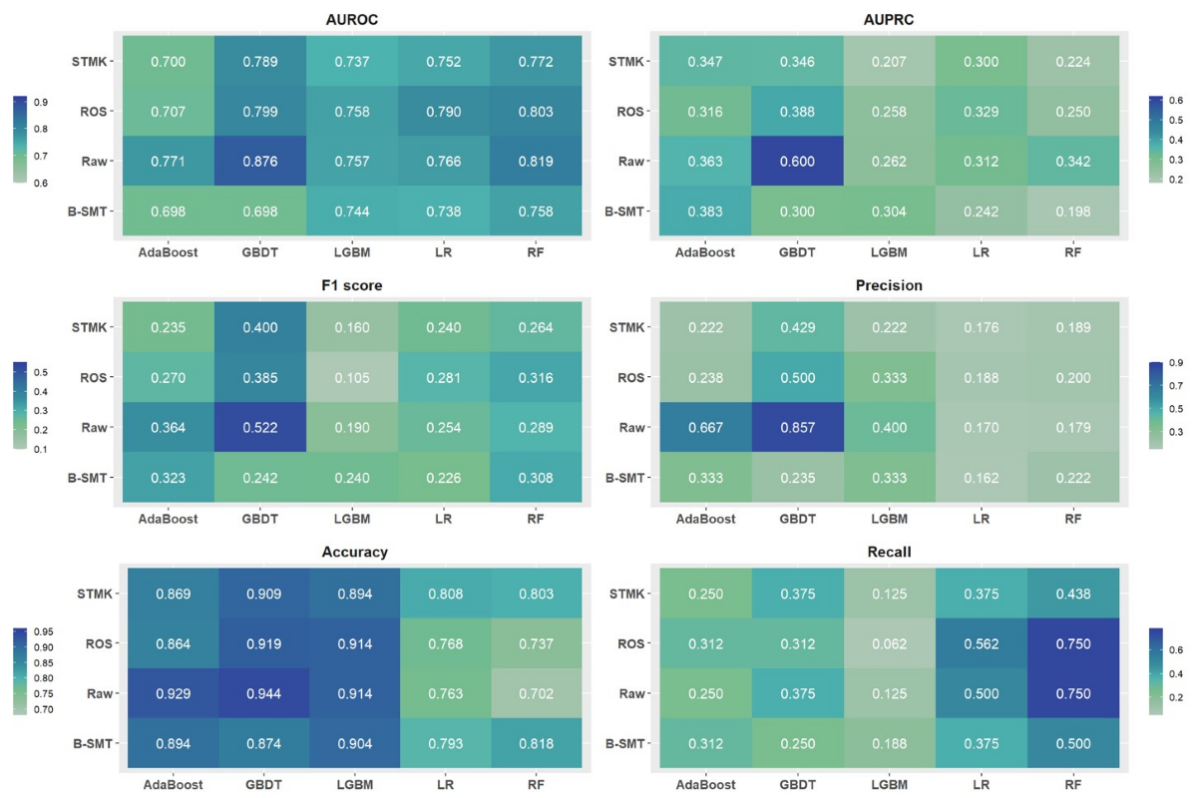
Model performance on temporal validation. The model performance evaluated by the six metrics is demonstrated in a heatmap. Blue and green colors represent higher and lower values, respectively. ROS: random oversampling; BSMT: Oversampling with Borderline Synthetic Minority Oversampling Technique; STMK: Synthetic Minority Oversampling Technique-Tomek Links; GBDT: Gradient Boosting Decision Tree; AdaBoost: Adaptive Boosting; LGBM: Light Gradient Boosting Machine; RF: random forest; LR: logistic regression; AUPRC: area under the precision-recall curve; AUROC: area under the receiver operating characteristic curve.

Figure 2 shows the model performance based on the AUPRC, AUROC, F1-score, precision, recall, and accuracy in 20 ML models of the temporal validation cohort. Most of the GBDT and AdaBoost models had higher AUPRC values and F1-scores, while recall was higher in the RF and LR models. Of all the ML models, the GBDT without resampling (GBDT_RAW) outperformed the other models, with an AUPRC of 0.600, AUROC of 0.876, and an F1-score of 0.522. The AUPRC of GBDT_RAW (0.600, 95% CI 0.350-0.798) was significantly higher than those of the GBDT_ROS (0.388, 95% CI 0.175‐0.618; *P*<.05), GBDT_BMST (0.300, 95% CI 0.124‐0.549; *P*<.05), and GBDT_STMK (0.346, 95% CI 0.147‐0.582; *P*<.05). Multimedia Appendix 8 lists model performances of 5-fold cross-validation with the training cohort and the statistical significance tests of the AUPRC of all the models.

### Threshold Adjustment

**Figure 3.**
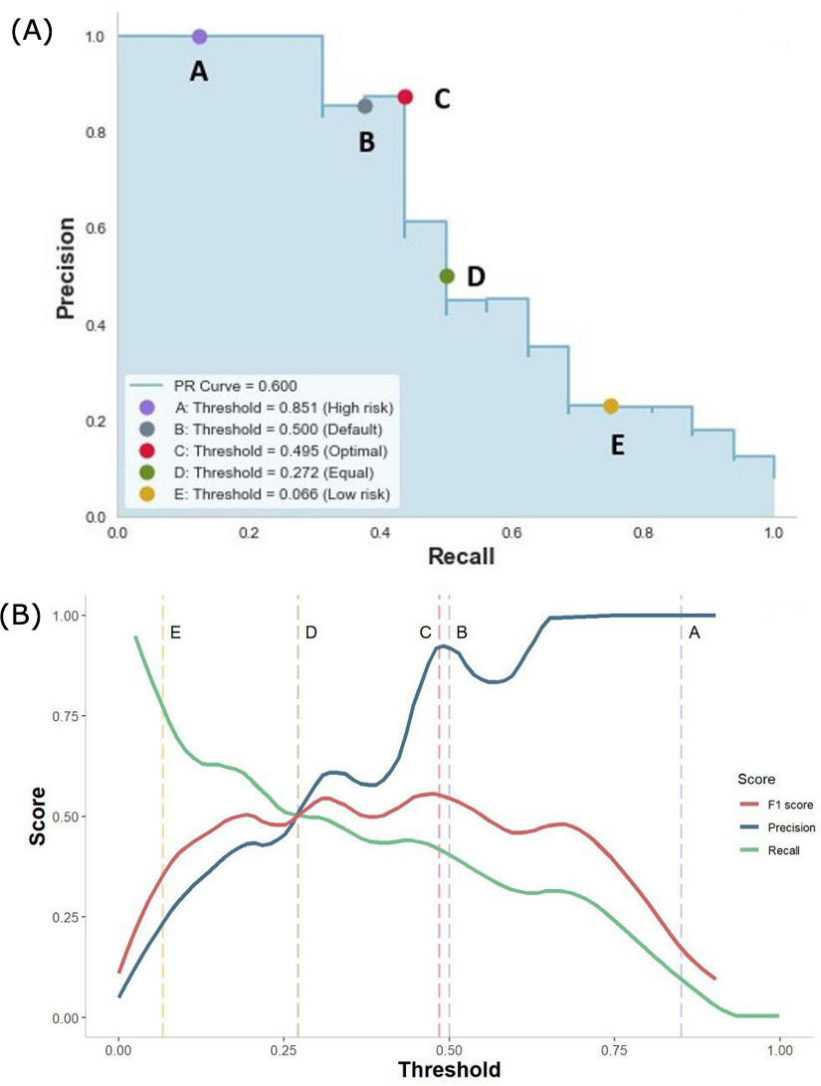
Threshold adjustment for the GBDT_RAW model. The five cutoff points on the precision-recall curve (A) represent different percentages of outcome predictions, precision, recall, and F1-scores based on different thresholds; (B) ,The X-axis represents the value of the threshold and the Y-axis shows values of the precision, recall, and F1-score.

Figure 3 shows the five cutoff points on the PRC and the precision, recall, and F1-score at different thresholds of the GBDT_RAW model. Based on different percentages of outcome predictions, cutoff points were 0.851 for identifying the top 1% of patients (2 predictive positive cases, point A — high-risk threshold),0.500 for the top 3.5% (7 predictive positive cases, point B — default threshold), 0.495 for the top 4% (8 predictive positive cases, point C — optimal threshold based on the maximum F1-score), 0.272 for the top 8% (16 predictive positive cases, point D — equal threshold), and 0.071 for the top 26% of patients (51 predictive positive cases, point E — low-risk threshold). When moving the threshold from 0.500 to 0.851 (point A), the precision increased from 0.875 to 1.000, while recall significantly decreased to 0.125. In contrast, recall significantly increased to 0.750 and the precision decreased to 0.231 when changing the threshold to 0.066 (point E). The precision, recall, and F1-score reached 0.500 when the threshold was adjusted to 0.272 (point D). The optimal threshold of 0.495 (point C) for the GBDT_RAW model was selected based on the maximum F1-score, with a precision of 0.875, recall of 0.438, and F1-score of 0.583.

### Model Interpretation

**Figure 4.**
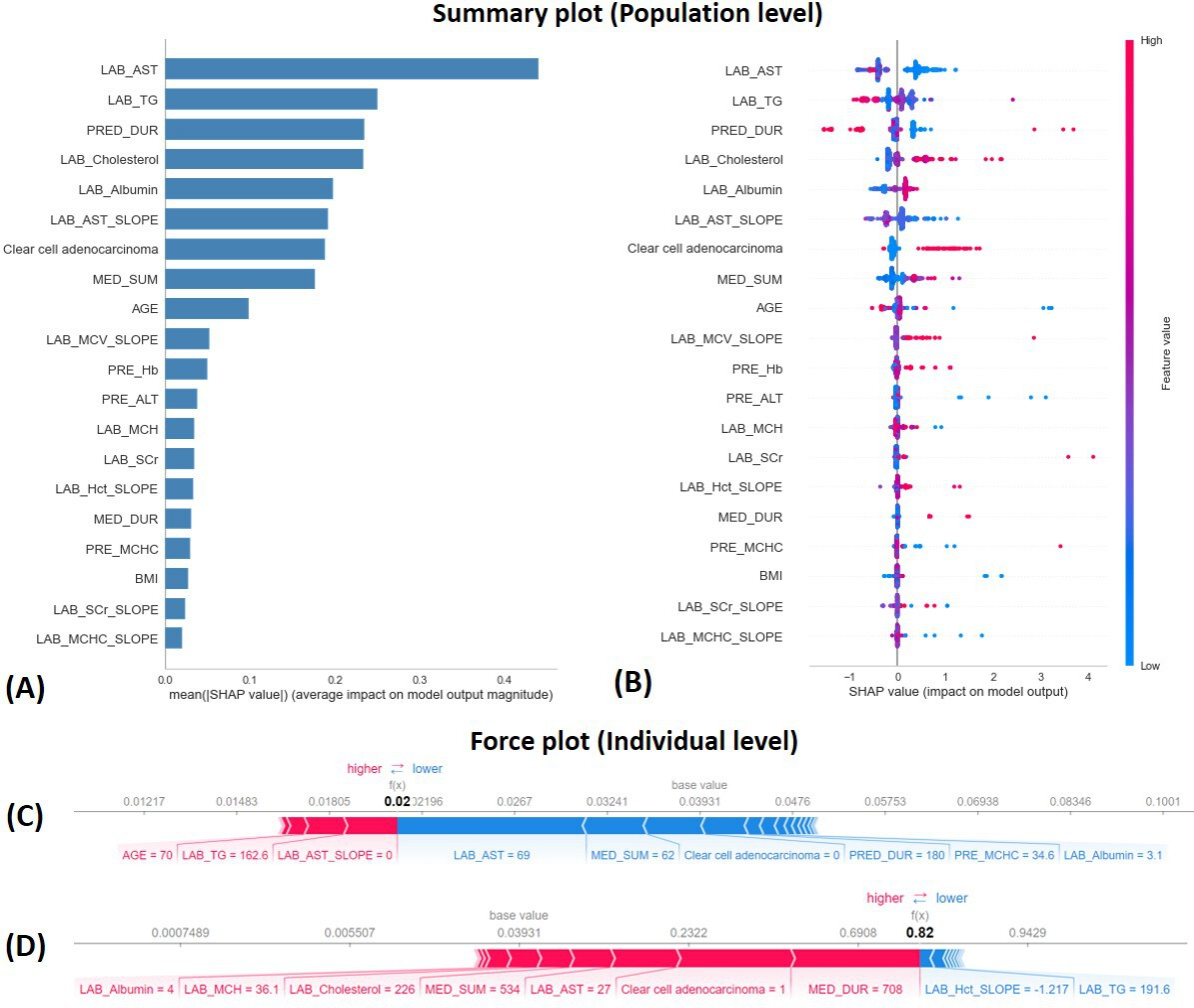
Model interpretation with a SHAP analysis for the GBDT_RAW model. At the population level, the summary bar plot (A) Mean SHAP values of all features, and a summary dot plot; (B) shows how each feature impacted the outcome prediction. The x-axis shows SHAP values of all features, and the colors represent the feature values, with red and blue respectively demonstrating higher and lower values. At the individual level, force plots in (C) and (D) show how features contributed to the model output value in each patient. Red and blue arrows represent positive and negative contributions of features, and the length of the arrow shows the magnitude of the impact on outcome predictions. LAB_AST: recent aspartate aminotransferase level; LAB_TG: recent triglyceride level; PRED_DUR: follow-up days; LAB_Cholesterol: recent cholesterol level; LAB_Albumin: recent albumin level; LAB_AST_SLOPE: slope of aspartate aminotransferase level; MED_SUM: sum days of medication; LAB_MCV_SLOPE: slope of mean corpuscular volume; PRE_Hb: previous hemoglobin level; PRE_ALT: previous alanine transaminase level; LAB_MCH: recent mean corpuscular haemoglobin level; LAB_SCr: recent serum creatinine level; LAB_Hct_SLOPE: slope of hematocrit level; MED_DUR: duration of medication; PRE_MCHC: previous mean corpuscular hemoglobin concentration level; LAB_SCr_SLOPE: slope of serum creatinine level; LAB_MCHC_SLOPE: slope of mean corpuscular hemoglobin concentration level.

Figure 4 shows model interpretation implemented with the SHAP analysis on the best-performing GBDT_RAW model. The summary plot in Figure 4A displays that the top five important predictors were the AST level, TG level, follow-up duration, cholesterol level, and albumin level. Figure 4B shows that patients with lower AST levels, higher cholesterol levels, higher albumin levels, longer durations of medication use, and clear cell adenocarcinoma histology had higher SHAP values for predicting the occurrence of thyroid dysfunction. At the individual level, force plots showed the impacts of features on predicting thyroid dysfunction in two specific patients. Figure 4C shows a patient with a predictive probability of 0.02, resulting from his AST level of 69 IU/L and the summed days of medications of 62 days, which contributed most negatively to outcome predictions. For a patient with a predictive probability of 0.82 in Figure 4D, the medication duration of 708 days and clear cell adenocarcinoma served as the most important factors that positively impacted outcome predictions.

### Web-Based Application

**Figure 5.**
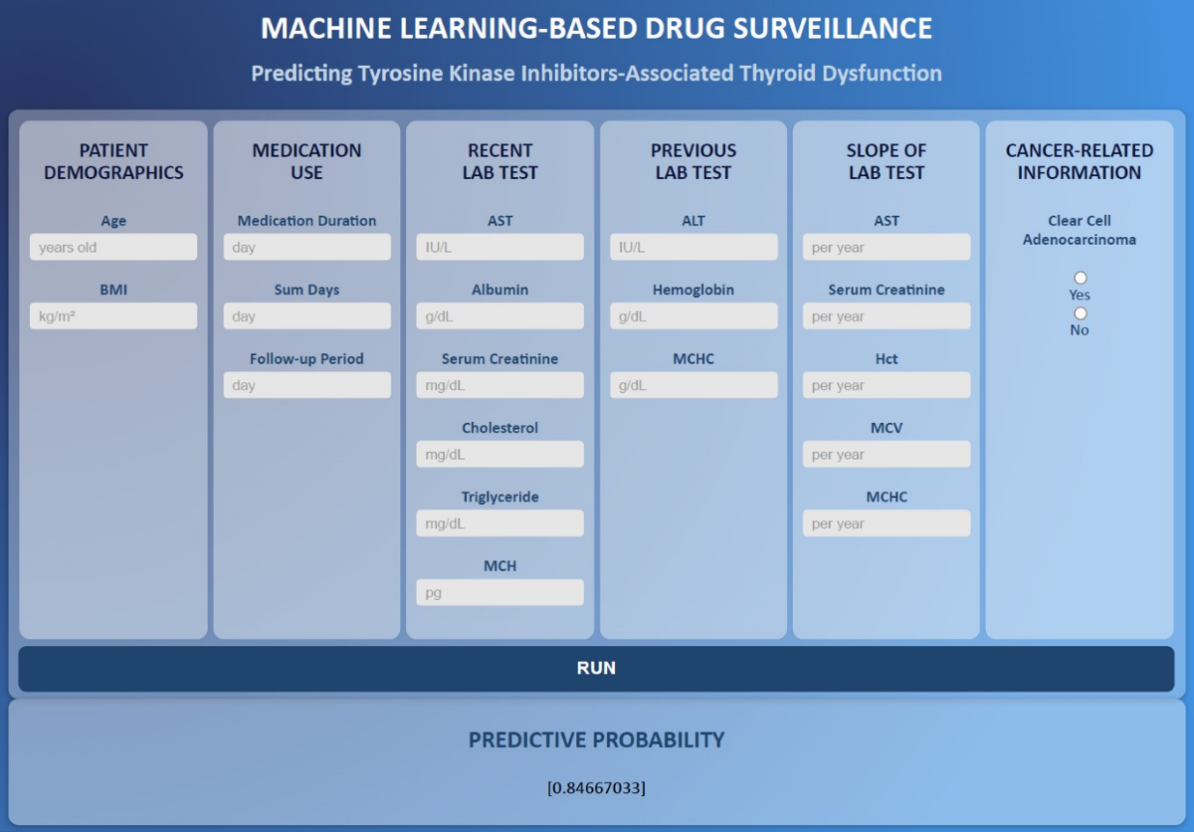
Web-based application with a user-friendly interface. A web-based application developed using the GBDT_RAW model showing 20 selected features selected and the predictive probability.

A web-based application for predicting sunitinib- and sorafenib-associated thyroid dysfunction was developed using the GBDT_RAW model (Figure 5). The application provides a user-friendly interface by showing 20 features selected by the GBDT model. Values of the features can be filled in with the appropriate units shown in each input box. The model then generates a predictive probability for the risk of sunitinib- or sorafenib-associated thyroid dysfunction. Ultimate predictions for the occurrence of thyroid dysfunction were determined based on thresholds adopted by clinical users.

## Discussion

### Principal Findings

This study developed explainable ML models by collecting time-series data to predict sunitinib- and sorafenib-associated thyroid dysfunction. The present model allowed ongoing predictions according to the rapidly shifting status of the disease for patients undergoing long-term TKI treatment. The best-performing GBDT without resampling model was optimized by threshold moving strategies to achieve a maximum F1-score. This study further used a SHAP analysis that provided interpretability at both the population and individual levels, identifying key predictors such as AST, cholesterol, and albumin levels. The feature selection process revealed similarities with prior research on amiodarone-induced thyroid dysfunction, supporting the model’s potential applicability to other drug-induced thyroid dysfunctions [9]. Integrating the model into a web-based application demonstrated its practical utility by allowing real-time risk estimation based on patient-specific data.

### Time-Series Data Collection

**Figure 6.**
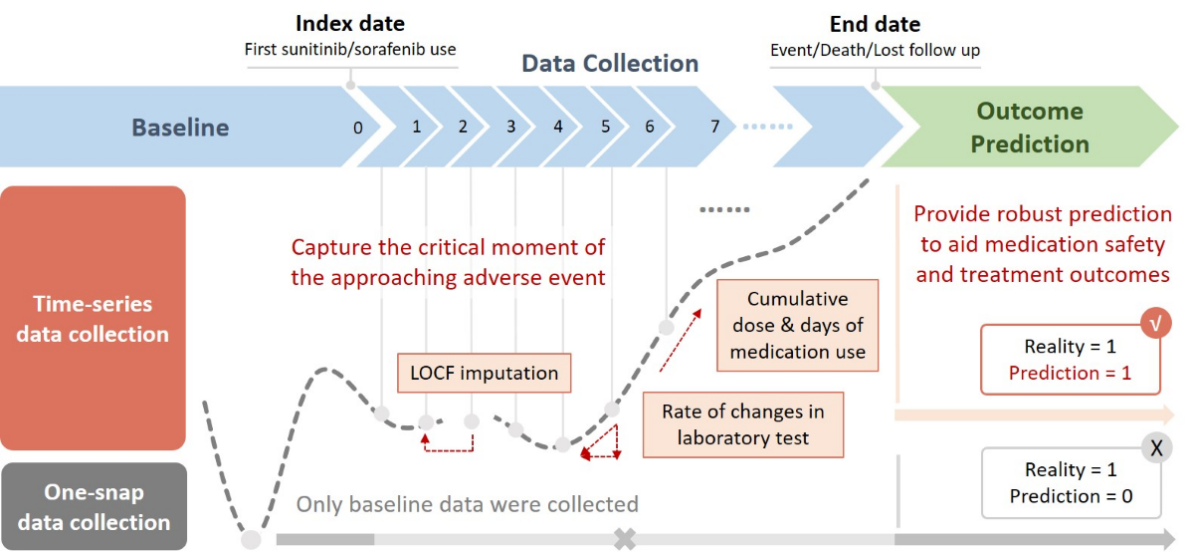
Graphic comparison of one-snap and time-series data collection methods. Time-series and one-snap data collection methods were compared. The dashed line represents possible changes in variables during a long-term follow-up period. LOCF: Last Observation Carried Forward

Time-series data collection in this study had a few advantages for thyroid dysfunction predictions on TKI users as illustrated in Figure 6. Clinical data at multiple time points in a long-term follow-up period were collected for model building. Time-series data allowed reengineering features by calculating rates of change of laboratory tests at recent and previous time points. The last observation carried forward method using the last value to replace missing data in subsequent time points ensured that missing values were imputed with plausible estimates [28]. This time-series data collection closely monitored patients newly prescribed TKI users and those on long-term TKI therapy. The time-series model captured the critical moment of an approaching adverse event for an individual patient, providing oncologists with invaluable information to treat patients on TKIs.

Identifying thyroid dysfunction was reported to potentially serve as a prognostic indicator for treatment outcomes. Sunitinib and sorafenib respectively demonstrated progression-free survival (PFS) periods of 11 and 5.5 months in patients with renal cell carcinoma [29,30]. Interestingly, studies indicated that patients with thyroid dysfunction had a longer PFS compared to patients with normal thyroid function among sunitinib (11.9 vs 8.8 months) and sorafenib users (19.3 vs 5.5 months) [4,31]. These findings suggest that thyroid dysfunction may not only represent an adverse event but could serve as a marker of enhanced therapeutic response. Leveraging a time-series data collection approach, this study can predict thyroid dysfunction risk while simultaneously identifying patients who may derive greater clinical benefits from sustained TKI therapy. Unlike one-snap data collection which arbitrarily differentiates high-risk patients from a low-risk group, this time-series approach allows high-risk patients to use lifesaving TKIs for a relatively longer grace period which can significantly enhance their survival times. Models with time-series data collection balance the risks and benefits of survival and adverse drug events, bestowing precious survival periods for end-stage cancer patients with reasonable medication safety and improved quality of life.

### Best-Performing Model With Threshold Adjustment

The GBDT_RAW model outperformed other machine learning models in this study, with thresholds adjusted to optimize model performance. The gradient boosting method analyzed nonlinear relationships and handled imbalanced data to predict diagnoses, hospital readmissions, and clinical outcomes [32-34]. During the hyperparameter tuning process, the AUPRC was considered, rather than the F1-score, without adjusting the threshold for imbalanced data [35,36]. The model without resampling outperformed resampling approaches by maintaining high precision, while the sparsity of the minority class in this study probably caused resampling to introduce noise or unrealistic synthetic data, failing to improve recall. The final model then used five cutoff points on the PRC to decide optimal thresholds and percentages of positive predictions. A relatively low threshold can identify all patients at risk but may increase the number of false positives, overwhelming clinicians with numerous warnings to review. On the contrary, choosing a relatively high threshold reduces the number of false positives but increases the chances of missing true positive cases. The selected threshold increased the precision, recall, and F1-score of the model’s performance, while considering the relative costs of false positives and false negatives to meet clinical needs [37].

### Model Interpretation and Potential for Model Expansion

Similar features for predicting drug-induced thyroid dysfunction were selected by the presence of TKIs and a previous amiodarone model built by our research team [9]. The feature selection process identified age, BMI, medication duration, summed days of medication, rate of change in serum creatinine levels, and recent mean corpuscular hemoglobin, AST, and cholesterol levels as key predictors of thyroid dysfunction in this study. Interestingly, these same features were also selected in our previous amiodarone model, suggesting their potential relevance across different drug-induced thyroid dysfunction models. Similar features highlight the potential for model expansion to increase generalizability. Top-ranked features identified in this study and their contributions calculated by the SHAP analysis were aligned with clinical domain knowledge. Higher cholesterol levels were found to positively impact predictions, which can be explained by the effect of thyroid function on lipoprotein metabolism [38]. The present study also found that longer summed days of medication increased the risk of thyroid dysfunction. This finding was confirmed by a long-term safety study of sunitinib, which showed the frequency of thyroid dysfunction, unlike other adverse events, gradually increased over time [39]. The AST level and histology of clear cell carcinoma served as important predictors, which may have resulted from a relatively lower incidence of thyroid dysfunction in patients with hepatocellular carcinoma and renal cell carcinoma.

### Clinical Implications of Web-Based Drug Surveillance

ML-based drug surveillance provides a promising tool for predicting and managing thyroid dysfunction caused by TKIs. Traditional clinical decision support systems (CDSS) rely on rule-based alerts for drug interactions or contraindications but often fail to capture the multifactorial nature of drug-induced thyroid dysfunction, which depends on thyroid-related factors [40]. AI-driven CDSSs, leveraging ML algorithms, can overcome these limitations by identifying complex patterns in patient data, providing personalized risk assessments, and generating timely alerts for thyroid function monitoring. Deploying such ML models on cloud-based platforms alongside rule-based CDSSs could enhance real-time risk stratification, enabling early detection, intervention, and potential adjustments in therapy to prevent endocrine complications. Further exploration of CDSSs incorporating ML predictions are warranted to improve clinical practice and medication safety [40].

### Limitations

There are a few limitations of this study. Data were from a single healthcare system and included limited numbers of sunitinib- or sorafenib-treated patients. There were missing data in the diagnosis of comorbidities and medication use. The nature of a retrospective study introduces underestimations of the incidence of thyroid dysfunction. As a result, different features selected by the present model and our previous amiodarone model mainly resulted from features with higher missing rates. Multicenter and multicountry studies for improving model extrapolation are needed before clinical application. Future qualitative research and prospective studies with the involvement of physicians should also be conducted to assess the usability and accessibility of the model for real-world evidence.

### Conclusions

This study applied time-series data collection to capture the critical moment of sunitinib- and sorafenib-associated thyroid dysfunction for ADR surveillance. The optimal threshold can balance the precision and recall meeting clinical needs. Feature importance was explained at the population and individual levels. The web-based application increased the model accessibility, allowing clinical users to receive real-time predictions. The comparison of features with amiodarone-induced thyroid dysfunction highlighted the potential for future model expansion.

## Supplementary material

10.2196/67767 Multimedia Appendix 1 Time-series data collection.

10.2196/67767 Multimedia Appendix 2 Codebook and missing rates of variables.

10.2196/67767 Multimedia Appendix 3 Hyperparameters tuning.

10.2196/67767 Multimedia Appendix 4 Pseudocodes of the model developing process.

10.2196/67767 Multimedia Appendix 5 Patient selection flowchart.

10.2196/67767 Multimedia Appendix 6 Patient characteristics and multivariate analysis.

10.2196/67767 Multimedia Appendix 7 Feature selection with recursive feature elimination.

10.2196/67767 Multimedia Appendix 8 Model performance of five-fold cross-validation.

## Abbreviations

AdaBoost: Adaptive Boosting
ADR: adverse drug reaction
ALT: alanine transaminase
AST: aspartate aminotransferase
AUPRC: area under the precision recall curve
AUROC: area under the receiver operating characteristic curve
CDSS: clinical decision support systems
CRD: clinical research database
GBDT: Gradient Boosting Decision Tree
LGBM: Light Gradient Boosting Machine
LR: logistic regression
ML: machine learning
RF: random forest
TKI: tyrosine kinase inhibitor
TMUH: Taipei Medical University Hospital
TRIPOD+AI: Transparent reporting of a multivariable prediction model for individual prognosis or diagnosis + artificial intelligence
TSH: thyroid-stimulating hormone
