# Supplementary material for: Web-Based Explainable Machine Learning-Based Drug Surveillance for Predicting Sunitinib- and Sorafenib-Associated Thyroid Dysfunction: Model Development and Validation Study
Source: JMIR Form Res. 2025 Apr 10;9:e67767. doi: 10.2196/67767 (PMC12005597; doi:10.2196/67767)
Supplement: Multimedia Appendix 1 [file formative-v9-e67767-s001.docx]

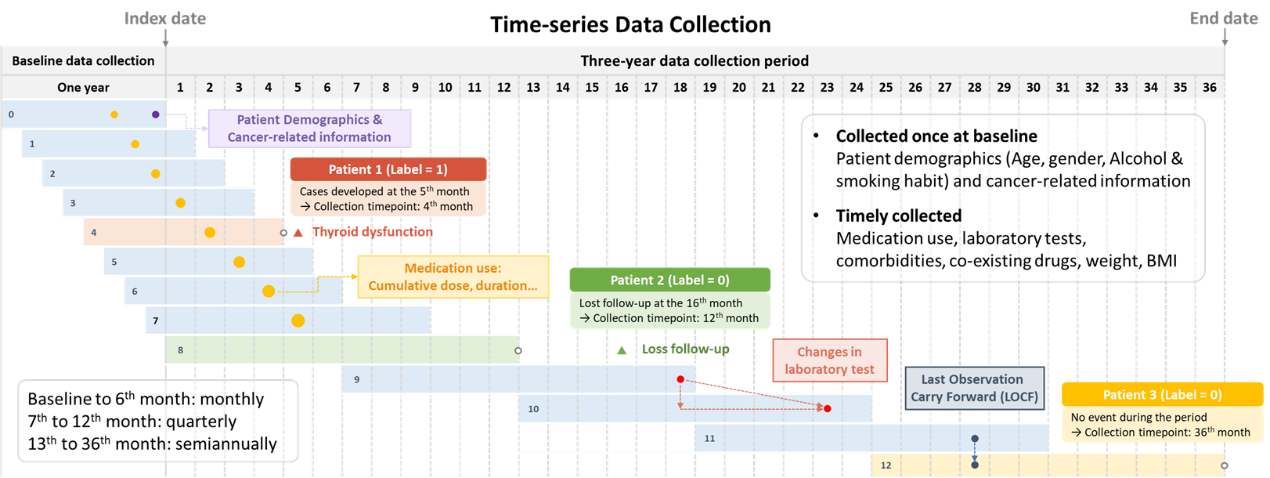


**Multimedia Appendix 1.** Time-series data collection

This figure shows a three-year data collection period with baseline and time-series data collection. The time points of data collection were at baseline, and in the 1st, 2nd, 3rd, 4th, 5th, 6th, 9th, 12th, 18th, 24th, 30th, and 36th months after the index date. When patients developed thyroid dysfunction cases or lost follow-up, data collection was stopped at the previous window. For example, patient 1 developed cases in the 5th month and thus was followed up until the 4th month. Patient 2 lost follow-up in the 16th month and thus was followed up until the 12th month. On the other hand, there was no thyroid adverse event for patient 3 during the follow-up period, and therefore, data collection was stopped in the 36th month.
