# Supplementary material for: Web-Based Explainable Machine Learning-Based Drug Surveillance for Predicting Sunitinib- and Sorafenib-Associated Thyroid Dysfunction: Model Development and Validation Study
Source: JMIR Form Res. 2025 Apr 10;9:e67767. doi: 10.2196/67767 (PMC12005597; doi:10.2196/67767)
Supplement: Multimedia Appendix 2 [file formative-v9-e67767-s002.docx]

**Multimedia Appendix 2.** Codebook and missing rates of variables

| **Variables** | **Full name** | **Type** | **Unit /Assignment** | **Missing rate**  **train/test (%)** |
| --- | --- | --- | --- | --- |
| AGE | Age at index date | Continuous | years old | 0/0 |
| SEX_TYPE | Gender | Categorical | 0: female; 1: male | 0/0 |
| WEIGHT | Weight | Continuous | kg | 0/0 |
| BMI | Body-mass index | Continuous | kg/m^2^ | - |
| SMOKE | Smoking habit | Categorical | 0: no; 1: yes | 0/0 |
| DRINK | Alcohol habit | Categorical | 0: no; 1: yes | 0/0 |
| CR_SITE_HCC | Hepatocellular carcinoma | Categorical | ICD 9: 155  ICD 10: C22 | 0/0 |
| CR_SITE_RCC | Renal cell carcinoma | Categorical | ICD 9: 189.0  ICD 10: C64 | 0/0 |
| CR_SITE_LUNG | Lung cancer | Categorical | ICD 9: 162  ICD 10: C33, C34 | 0/0 |
| CR_STAGE | Cancer stage | Categorical | 1, 2, 3, 4 | 30.67/47.25 |
| Hepatocellular  carcinoma | Hepatocellular carcinoma | Categorical | ICD-0-3: 817 | 0/0 |
| Clear cell  adenocarcinoma | Clear cell adenocarcinoma | Categorical | ICD-0-3: 831 | 0/0 |
| Adenocarcinoma | Adenocarcinoma | Categorical | ICD-0-3: 814 | 0/0 |
| MED_CODE | Medication | Categorical | 1: Sunitinib;  0: Sorafenib | 0/0 |
| MED_DUR | Duration of medication | Continuous | days | 0/0 |
| MED_SUM | Sum days of medication | Continuous | days | 0/0 |
| CUM_SU | Cumulative dose (Sunitinib) | Continuous | mg | 0/0 |
| CUM_SO | Cumulative dose (Sorafenib) | Continuous | mg | 0/0 |
| DOSE_SU | Sunitinib dose | Continuous | mg | 0/0 |
| DOSE_SO | Sorafenib dose | Continuous | mg | 0/0 |
| PRED_DUR | Follow-up days | Continuous | days | 0/0 |
| DOSE_ADJ | Dose reduction | Categorical | 0: Initial; 1: First;  2: Second | 50.08/63.64 |
| DX_htn | Hypertension | Categorical | ICD 9: 401, 402, 403,  404, 405  ICD 10: I10, I11, I.12, I13, I14, I15, N26.2 | 0/0 |
| DX_dm | Diabetes | Categorical | ICD 9: 250  ICD 10: E08, E09, E10, E11, E12, E13, E14 | 0/0 |
| DX_lipid | Hyperlipidemia | Categorical | ICD 9: 272.0, 272.1, 272.2, 272.3, 272.4  ICD 10: E78.0, E78.1, E78.2, E78.3, E78.4, E78.5 | 0/0 |
| DX_lc | Liver cirrhosis | Categorical | ICD 9: 571.2, 571.5, 571.6  ICD 10: K70.2, K70.30, K70.31, K74.0, K74.1, K74.2, K74.3, K74.4, K74.5, K74.60, K74.69 | 0/0 |
| DX_ckd | CKD^a^ | Categorical | ICD 9: 585  ICD 10: N18.4, N18.5,  N18.6, N18.9 | 0/0 |
| DX_anemia | Anemia | Categorical | ICD 9: 280, 281, 282, 283, 284, 285  ICD 10: D50, D51, D52, D53, D55, D57, D58, D59, D60, D61, D63, D64 | 0/0 |
| DX_gout | Gout | Categorical | ICD 9: 274  ICD 10: M10 | 0/0 |
| DX_ppi | PPI^b^ | Categorical | ATC_CODE: A02BC | 0/0 |
| DX_nsaids | NSAIDs^c^ | Categorical | ATC_CODE: M01A | 0/0 |
| DX_epileptic | Antiepileptic drugs | Categorical | ATC_CODE: N03A | 0/0 |
| DX_psychotic | Antipsychotic drugs | Categorical | ATC_CODE: N05A | 0/0 |
| DX_beta | Beta-blocker | Categorical | ATC_CODE: C07A | 0/0 |
| DX_metformin | Metformin | Categorical | ATC_CODE: A10BA02 | 0/0 |
| DX_AntiDM | AntiDM^d^ | Categorical | ATC_CODE: A10BB01, A10BB04, A10BB07, A10BB08, A10BB09, A10BB12, A10BD01, A10BD02, A10BG02, A10BG03, A10BD07, A10BD08, A10BD13, A10BH01, A10BH02, A10BH03, A10BH04, A10BH05 | 0/0 |
| DX_cyp3a4 | CYP3A4 inhibitors | Categorical | ATC_CODE: J05AE03, J02AC02, J02AB02, J05AR10, J05AE02, J05AP53, J02AC04, J05AE01, J05AE09, J01FA15, J01FA08, J02AC03, A04AD12, J01MA02, C03XA02, L01ED01, C05AE03, C01BD07, J01FA01, J02AC01, N06AB08, L01EA01, J02AC05, N05BA23, C08DA01 | 0/0 |
| DX_thyroid | Thyroid-related drugs | Categorical | ATC_CODE: C01BD01, D08AG02, L01XX23, L01FF,  L01FX04, N05AN01, L03AC01, L03AB01, L03AB04, L03AB05, L03AB11, L03AB10, L04AA34, G03C, L02BA01, G03XC01, N07BC02, N07BC06, L01BC02, G03B, C10AC01, C10AC02, H03B, A02BX02, H01CB02, C03CA01 | 0/0 |
| LAB_TSH | Recent TSH^e^ | Continuous | uIU/mL | 89.69/95.94 |
| LAB_AST | Recent AST^f^ | Continuous | IU/L | 0.93/3.80 |
| LAB_ALT | Recent ALT^g^ | Continuous | IU/L | 1.28/4.23 |
| LAB_SCr | Recent SCr^h^ | Continuous | mg/dL | 0.36/2.96 |
| LAB_Albumin | Recent Albumin | Continuous | g/dL | 11.07/35.59 |
| LAB_Bilirubin | Recent Bilirubin | Continuous | mg/dL | 9.90/2.22 |
| LAB_Cholesterol | Recent Cholesterol | Continuous | mg/dL | 66.79/74.47 |
| LAB_TG | Recent TG^i^ | Continuous | mg/dL | 65.72/74.89 |
| LAB_RBC | Recent RBC^j^ | Continuous | 10^6^/uL | 1.12/3.80 |
| LAB_Hb | Recent Hb^k^ | Continuous | g/dL | 0.96/3.04 |
| LAB_Hct | Recent Hct^l^ | Continuous | % | 1.12/3.80 |
| LAB_MCV | Recent MCV^m^ | Continuous | fL | 1.12/3.89 |
| LAB_MCHC | Recent MCHC^n^ | Continuous | g/dL | 1.31/3.89 |
| LAB_MCH | Recent MCH^o^ | Continuous | pg | 1.31/3.89 |
| PRE_AST | Previous AST | Continuous | IU/L | 1.28/5.24 |
| PRE_ALT | Previous ALT | Continuous | IU/L | 1.75/5.75 |
| PRE_SCr | Previous SCr | Continuous | mg/dL | 0.57/4.31 |
| PRE_Bilirubin | Previous Bilirubin | Continuous | mg/dL | 11.45/25.27 |
| PRE_RBC | Previous RBC | Continuous | 10^6^/uL | 1.37/5.16 |
| PRE_Hb | Previous Hb | Continuous | g/dL | 1.12/4.40 |
| PRE_Hct | Previous Hct | Continuous | % | 1.37/5.16 |
| PRE_MCV | Previous MCV | Continuous | fL | 1.37/5.33 |
| PRE_MCHC | Previous MCHC | Continuous | g/dL | 1.67/5.33 |
| PRE_MCH | Previous MCH | Continuous | pg | 1.67/5.33 |
| LAB_AST_SLOPE | Slope of AST | Continuous | $\frac{\mathrm{AST}_{-1}-\mathrm{AST}_{-2}}{Day interval}*365$ | 13.63/18.69 |
| LAB_ALT_SLOPE | Slope of ALT | Continuous | $\frac{\mathrm{ALT}_{-1}-\mathrm{ALT}_{-2}}{Day interval}*365$ | 13.79/19.19 |
| LAB_SCr_SLOPE | Slope of SCr | Continuous | $\frac{\mathrm{SCr}_{-1}-\mathrm{SCr}_{-2}}{Day interval}*365$ | 13.46/19.19 |
| LAB_Bilirubin_SLOPE | Slope of Bilirubin | Continuous | $\frac{\mathrm{Bilirubin}_{-1}-\mathrm{Bilirubin}_{-2}}{Day interval}*365$ | 13.96/19.19 |
| LAB_RBC_SLOPE | Slope of RBC | Continuous | $\frac{\mathrm{RBC}_{-1}-\mathrm{RBC}_{-2}}{Day interval}*365$ | 13.96/19.19 |
| LAB_Hb_SLOPE | Slope of Hb | Continuous | $\frac{\mathrm{Hb}_{-1}-\mathrm{Hb}_{-2}}{Day interval}*365$ | 13.96/19.19 |
| LAB_Hct_SLOPE | Slope of Hct | Continuous | $\frac{\mathrm{Hct}_{-1}-\mathrm{Hct}_{-2}}{Day interval}*365$ | 13.96/19.19 |
| LAB_MCV_SLOPE | Slope of MCV | Continuous | $\frac{\mathrm{MCV}_{-1}-\mathrm{MCV}_{-2}}{Day interval}*365$ | 13.96/19.19 |
| LAB_MCHC_SLOPE | Slope of MCHC | Continuous | $\frac{\mathrm{MCHC}_{-1}-\mathrm{MCHC}_{-2}}{Day interval}*365$ | 13.96/19.19 |
| LAB_MCH_SLOPE | Slope of MCH | Continuous | $\frac{\mathrm{MCH}_{-1}-\mathrm{MCH}_{-2}}{Day interval}*365$ | 13.96/19.19 |
| hypo | Thyroid dysfunction | Categorical | 0: no; 1: yes | 0 |

^a^CKD: Chronic Kidney Disease

^b^PPI: Proton-pump inhibitor

^c^NSAIDs: Non-steroidal anti-inflammatory drugs

^d^AntiDM: Antidiabetic drugs

^e^TSH: Thyroid stimulating hormone

^f^AST: Aspartate aminotransferase

^g^ALT: Alanine transaminase

^h^SCr: Serum creatinine

^i^TG: Triglyceride

^j^RBC: Red blood cell

^k^Hb: Hemoglobin

^l^Hct: Hematocrit

^m^MCV: Mean corpuscular volume

^n^MCHC: Mean corpuscular haemoglobin concentration

^o^MCH: Mean corpuscular haemoglobin
