# Supplementary material for: Web-Based Explainable Machine Learning-Based Drug Surveillance for Predicting Sunitinib- and Sorafenib-Associated Thyroid Dysfunction: Model Development and Validation Study
Source: JMIR Form Res. 2025 Apr 10;9:e67767. doi: 10.2196/67767 (PMC12005597; doi:10.2196/67767)
Supplement: Multimedia Appendix 3 [file formative-v9-e67767-s003.docx]

**Table S3.1.** Hyperparameters tuning with Bayesian optimization

| **Algorithm** | **Hyperparameters** |
| --- | --- |
| **GBDT^a^** | learning_rate: (0.01,1)  n_estimators: (100,1000) |
| **AdaBoost** | n_estimators: (100,1000)  learning_rate: (0.01, 1.0)  algorithm: ('SAMME', 'SAMME.R') |
| **LGBM^b^** | learning_rate: (0.01,1)  n_estimators: (100,1000)  subsample: (0.8,1.0)  colsample_bytree: (0.8,1.0) |
| **Random forest** | bootstrap: (True, False)  n_estimators: (100, 1000)  min_samples_split: (2, 10)  min_samples_leaf: (1,5)  max_depth: (3,7) |

**Table S3.2.** The final best hyperparameters of each model

| **GBDT** | | | | |
| --- | --- | --- | --- | --- |
|  | **RAW** | **ROS^c^** | **BSMT^d^** | **STMK^e^** |
| learning_rate | 0.07010 | 0.87767 | 0.98513 | 1.0 |
| n_estimators | 100 | 221 | 100 | 981 |
| **AdaBoost** | | | | |
|  | **RAW** | **ROS** | **BSMT** | **STMK** |
| algorithm | SAMME | SAMME.R | SAMME.R | SAMME.R |
| learning_rate | 1.0 | 1.0 | 0.34605 | 1.0 |
| n_estimators | 607 | 100 | 996 | 886 |
| **LGBM** | | | | |
|  | **RAW** | **ROS** | **BSMT** | **STMK** |
| colsample_bytree | 0.92676 | 0.87573 | 0.82015 | 0.8 |
| learning_rate | 0.01 | 0.63630 | 0.16766 | 0.41090 |
| n_estimators | 970 | 107 | 198 | 240 |
| subsample | 0.98953 | 0.88839 | 0.82517 | 0.8 |
| **Random forest** | | | | |
|  | **RAW** | **ROS** | **BSMT** | **STMK** |
| bootstrap | False | False | True | False |
| max_depth | 3 | 3 | 7 | 7 |
| min_samples_leaf | 1 | 1 | 1 | 4 |
| min_samples_split | 6 | 2 | 2 | 7 |
| n_estimators | 100 | 382 | 1000 | 305 |

^a^GBDT: Gradient Boosting Decision Tree

^b^LiGBM: Light Gradient Boosting Machine

^c^ROS: Random oversampling

^d^BSMT: Oversampling with Borderline Synthetic Minority Oversampling Technique

^e^STMK: Synthetic Minority Oversampling Technique-Tomek Links
