# Supplementary material for: Web-Based Explainable Machine Learning-Based Drug Surveillance for Predicting Sunitinib- and Sorafenib-Associated Thyroid Dysfunction: Model Development and Validation Study
Source: JMIR Form Res. 2025 Apr 10;9:e67767. doi: 10.2196/67767 (PMC12005597; doi:10.2196/67767)
Supplement: Multimedia Appendix 4 [file formative-v9-e67767-s004.docx]

**Multimedia Appendix 4.** Pseudocodes of the model developing process

| **Process** | **Pseudocode** |
| --- | --- |
| Bayesian optimization | 1. Create the machine learning models   For logistic regression, random forest, GBDT, LGBM, and AdaBoost:  classifier = LogisticRegression(initialized parameters)  classifier = RandomForestClassifier(initialized parameters)  classifier = GradientBoostingClassifier(initialized parameters)  classifier = lgb.LGBMClassifier(initialized parameters)  classifier = AdaBoostClassifier (initialized parameters)   1. Create the parameters grid   search_spaces = {Put ranges of hyperparameters}  train_x = All the interested features  train_y = Prediction outcome  kfold = StratifiedKFold(Split the data into 5 groups)  bayes_imba = BayesSearchCV(put into the classifier, search_spaces and kfold)  bayes_imba.fit(train_x, train_y)   1. Calculate the best average precision with the best combination of hyperparameters |
| Recursive  feature elimination (RFE) | 1. Create the RFE object and compute a cross-validated score.   rfecv = RFECV(classifier, StratifiedKFold(Split the data into 5 groups))   1. train_x = All the interested features   train_y = Prediction outcome  rfecv.fit(train_x, train_y)   1. Select the best roc_auc collection of features   print("Optimal number of features for model")  print("selected features for model") |
| Five-fold cross-validation | 1. StratifiedKFold(Split the data into 5 groups) 2. Load the optimized model by Bayesian optimization with the best combination of features by RFE. 3. Calculate the accuracy, precision, recall, F1 score, AUROC, and AUPRC of the model. |
