# Supplementary material for: Web-Based Explainable Machine Learning-Based Drug Surveillance for Predicting Sunitinib- and Sorafenib-Associated Thyroid Dysfunction: Model Development and Validation Study
Source: JMIR Form Res. 2025 Apr 10;9:e67767. doi: 10.2196/67767 (PMC12005597; doi:10.2196/67767)
Supplement: Multimedia Appendix 5 [file formative-v9-e67767-s005.docx]

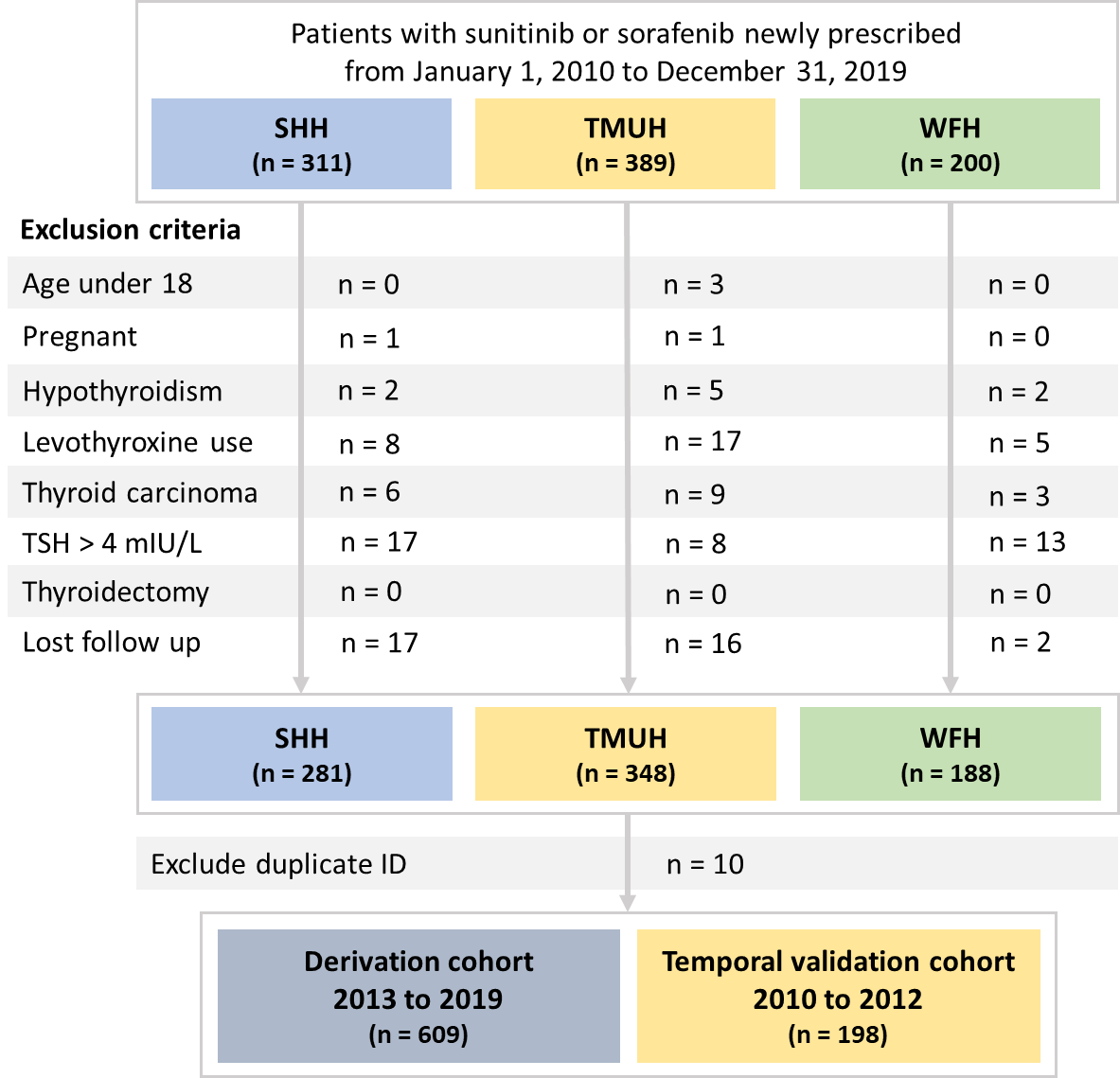


**Multimedia Appendix 5.** Patient selection flowchart.

*SHH: Shuang Ho Hospital; TMUH: Taipei Medical University Hospital; WFH: Wan Fang Hospital
