# Supplementary material for: Web-Based Explainable Machine Learning-Based Drug Surveillance for Predicting Sunitinib- and Sorafenib-Associated Thyroid Dysfunction: Model Development and Validation Study
Source: JMIR Form Res. 2025 Apr 10;9:e67767. doi: 10.2196/67767 (PMC12005597; doi:10.2196/67767)
Supplement: Multimedia Appendix 6 [file formative-v9-e67767-s006.docx]

**Multimedia Appendix 6.** Patient characteristics and multivariate analysis

|  | **Derivation cohort (n = 609)** | | | **Temporal validation cohort (n = 198)** | | |
| --- | --- | --- | --- | --- | --- | --- |
|  | TD^a^ (n = 52) | Non-TD | *P-value* | TD (n = 16) | Non-TD | *P-value* |
| **Demographics** |  |  |  |  |  |  |
| Age, median (IQR) | 64.00 (12.25) | 64.00 (16.00) | 0.587 | 58.50 (21.50) | 61.00 (19.00) | 0.686 |
| Gender (male), n (%) | 35 (67.31) | 398 (71.45) | 0.638 | 9 (56.25) | 117 (64.29) | 0.712 |
| Weight (kg),  median (IQR) | 62.60 (15.20) | 61.50 (14.00) | 0.605 | 64.61 (13.58) | 63.00 (10.08) | 0.051 |
| BMI^b^, median (IQR) | 22.98 (4.79) | 23.39 (4.27) | 0.773 | 24.14 (4.15) | 23.41 (1.21) | 0.672 |
| Smoking, n (%) | 10 (19.23) | 163 (29.26) | 0.170 | 0 (0.00) | 29 (15.93) | 0.135 |
| Alcohol, n (%) | 13 (25.00) | 124 (22.26) | 0.781 | 1 (6.25) | 21 (11.54) | 1.000 |
| **Cancer type, n (%)** |  |  |  |  |  |  |
| HCC^c^ | 25 (48.08) | 396 (71.10) | <.001 | 3 (18.75) | 97 (53.30) | <.001 |
| RCC^d^ | 17 (32.69) | 59 (10.59) |  | 7 (43.75) | 13 (7.14) |  |
| Lung cancer | 2 (3.85) | 29 (5.21) |  | 0 (0.00) | 23 (12.64) |  |
| **Histology, n (%)** |  |  |  |  |  |  |
| Hepatocellular carcinoma | 22 (42.31) | 367 (65.89) | <.001 | 2 (12.50) | 87 (47.80) | <.001 |
| Clear cell adenocarcinoma | 15 (28.85) | 43 (7.72) |  | 7 (43.75) | 12 (6.59) |  |
| Adenocarcinoma | 1 (1.92) | 20 (3.59) |  | 0 (0.00) | 24 (13.19) |  |
| **Medication use** |  |  |  |  |  |  |
| Sunitinib, n (%) | 22 (42.31) | 142 (25.49) | 0.014 | 11 (68.75) | 79 (43.41) | 0.091 |
| Sorafenib, n (%) | 30 (57.69) | 415 (74.51) | 0.014 | 5 (31.25) | 103 (56.59) | 0.091 |
| Duration (day),  median (IQR) | 78.50 (232.50) | 60.00 (119.00) | 0.467 | 234.50 (379.75) | 68.50 (143.75) | 0.208 |
| Sum days (day),  median (IQR) | 70.50 (177.50) | 56.00 (88.00) | 0.301 | 108.50 (257.00) | 48.97 (91.00) | 0.268 |
| Sunitinib dose  (mg/day), median (IQR) | 37.50 (12.50) | 25.00 (25.00) | 0.008 | 37.50 (12.50) | 25.00 (0.00) | 0.035 |
| Sorafenib dose  (mg/day), median (IQR) | 600.00 (400.00) | 600.00 (400.00) | 0.564 | 400.00 (600.00) | 400.00 (400.00) | 0.817 |
| **Comorbidities, n (%)** |  |  |  |  |  |  |
| Hypertension | 18 (34.62) | 162 (29.08) | 0.498 | 8 (50.00) | 41 (22.53) | 0.032 |
| Diabetes | 9 (17.31) | 120 (21.54) | 0.591 | 9 (56.25) | 28 (15.38) | <.001 |
| Hyperlipidemia | 5 (9.62) | 57 (10.23) | 1.000 | 4 (25.00) | 8 (4.40) | 0.010 |
| CKD^e^ | 5 (9.62) | 19 (3.41) | 0.068 | 4 (25.00) | 6 (3.30) | 0.005 |
| Anemia | 3 (5.77) | 44 (7.90) | 0.787 | 1 (6.25) | 23 (12.64) | 0.699 |
| **Co-existing drugs, n (%)** |  |  |  |  |  |  |
| PPI^f^ | 20 (38.46) | 283 (50.81) | 0.119 | 2 (12.50) | 79 (43.41) | 0.017 |
| Antiepileptic drugs | 10 (19.23) | 110 (19.75) | 1.000 | 4 (25.00) | 28 (15.38) | 0.299 |
| Antipsychotic drugs | 17 (32.69) | 198 (35.55) | 0.795 | 1 (6.25) | 58 (31.87) | 0.043 |
| Thyroid-related drugs |  |  |  |  |  |  |
| 0 | 26 (50.00) | 197 (35.37) | 0.110 | 6 (37.50) | 57 (31.32) | 0.401 |
| 1 | 17 (32.69) | 241 (43.27) |  | 9 (56.25) | 87 (47.80) |  |
| ≧2 | 9 (17.31) | 119 (21.36) |  | 1 (6.25) | 38 (20.88) |  |
| **Lab tests, median (IQR)** |  |  |  |  |  |  |
| TSH^g^ (uIU/mL) | 1.89 (1.08) | 2.17 (0.97) | 0.001 | 1.62 (0.76) | 2.20 (0.95) | <.001 |
| AST^h^ (IU/L) | 28.50 (20.75) | 57.00 (78.00) | <.001 | 31.70 (17.50) | 59.00 (81.25) | 0.001 |
| ALT^i^ (IU/L) | 24.51 (22.50) | 38.00 (46.00) | <.001 | 22.50 (21.45) | 36.00 (40.50) | 0.027 |
| SCr^j^ (mg/dL) | 0.95 (0.76) | 0.89 (0.49) | 0.111 | 1.30 (1.44) | 0.90 (0.47) | 0.002 |
| Albumin (g/dL) | 3.70 (0.75) | 3.50 (1.00) | 0.043 | 3.80 (0.39) | 3.50 (0.90) | <.001 |
| Bilirubin  (mg/dL) | 0.79 (0.63) | 1.10 (1.40) | <.001 | 0.63 (0.77) | 1.17 (1.44) | <.001 |
| Cholesterol  (mg/dL) | 179.97 (77.80) | 172.00 (58.65) | 0.877 | 175.09 (51.30) | 171.62 (67.54) | 0.828 |
| TG^k^ (mg/dL) | 103.51 (43.95) | 108.00 (70.55) | 0.940 | 115.96 (75.04) | 101.55 (67.52) | 0.150 |
| RBC^l^ (10^6^/uL) | 3.79 (1.06) | 3.70 (1.05) | 0.894 | 3.20 (0.99) | 3.35 (1.13) | 0.648 |
| Hb^m^ (g/dL) | 11.30 (2.88) | 11.50 (3.20) | 0.782 | 9.80 (2.65) | 10.75 (2.77) | 0.282 |
| Hct^n^ (%) | 33.75 (8.55) | 34.00 (9.50) | 0.573 | 30.50 (7.03) | 30.70 (8.83) | 0.843 |

^a^TD: Thyroid dysfunction

^b^BMI: Body mass index

^c^HCC: Hepatocellular carcinoma

^d^RCC: Renal cell carcinoma

^e^CKD: Chronic Kidney Disease

^f^PPI: Proton-pump inhibitor

^g^TSH: Thyroid stimulating hormone

^h^AST: Aspartate aminotransferase

^i^ALT: Alanine transaminase

^j^SCr: Serum creatinine

^k^TG: Triglyceride

^l^RBC: Red blood cell

^m^Hb: Hemoglobin

^n^Hct: Hematocrit
