# Supplementary material for: Web-Based Explainable Machine Learning-Based Drug Surveillance for Predicting Sunitinib- and Sorafenib-Associated Thyroid Dysfunction: Model Development and Validation Study
Source: JMIR Form Res. 2025 Apr 10;9:e67767. doi: 10.2196/67767 (PMC12005597; doi:10.2196/67767)
Supplement: Multimedia Appendix 7 [file formative-v9-e67767-s007.docx]

**Multimedia Appendix 7.** Feature selection with recursive feature elimination

| **Features**  **(number)** | **GBDT**  **(n = 20)** | **AdaBoost**  **(n = 18)** | **LGBM**  **(n = 15)** | **RF**  **(n = 40)** | **LR**  **(n = 20)** |
| --- | --- | --- | --- | --- | --- |
| **Demographics** |  |  |  |  |  |
| Age | V | V | V | V |  |
| Gender |  |  |  |  | V |
| Weight |  |  | V | V | V |
| BMI^a^ | V | V | V | V | V |
| Smoking |  |  |  |  | V |
| Alcohol |  |  |  |  | V |
| **Cancer-related information** |  |  |  |  |  |
| HCC^b^ |  |  |  |  |  |
| RCC^c^ |  |  |  |  |  |
| Lung cancer |  |  |  |  |  |
| Cancer stage |  |  |  |  |  |
| Hepatocellular carcinoma |  |  |  |  | V |
| Clear cell adenocarcinoma | V |  |  |  | V |
| Adenocarcinoma |  |  |  |  |  |
| **Medication use** |  |  |  |  |  |
| Medication |  |  |  |  | V |
| Duration of medication | V |  |  | V |  |
| Sum days of medication | V | V | V | V |  |
| Cumulative dose (Sunitinib) |  |  |  |  |  |
| Cumulative dose (Sorafenib) |  |  |  | V |  |
| Sunitinib dose |  |  |  | V |  |
| Sorafenib dose |  |  |  |  |  |
| Follow-up days | V | V |  | V | V |
| Dose reduction |  |  |  |  |  |
| **Comorbidities** |  |  |  |  |  |
| Hypertension |  |  |  |  |  |
| Diabetes |  |  |  |  |  |
| Hyperlipidemia |  |  |  |  |  |
| Liver cirrhosis |  |  |  |  |  |
| CKD^d^ |  |  |  |  |  |
| Anemia |  |  |  |  | V |
| Gout |  |  |  |  |  |
| **Co-existing drugs** |  |  |  |  |  |
| PPI^e^ |  |  |  |  |  |
| NSAIDs^f^ |  |  |  |  |  |
| Antiepileptic |  |  |  |  |  |
| Antipsychotic |  |  |  |  |  |
| Beta-blocker |  |  |  |  |  |
| Metformin |  |  |  |  |  |
| AntiDM^g^ |  |  |  |  |  |
| CYP3A4 inhibitors |  |  |  |  |  |
| Thyroid-related drugs |  |  |  |  | V |
| **Recent lab test** |  |  |  |  |  |
| TSH^h^ |  | V | V | V |  |
| AST^i^ | V | V | V | V | V |
| ALT^j^ |  | V | V | V | V |
| SCr^k^ | V | V |  | V |  |
| Albumin | V |  |  | V |  |
| Bilirubin |  | V | V | V | V |
| Cholesterol | V | V | V | V |  |
| TG^l^ | V | V | V | V |  |
| RBC^m^ |  |  |  | V | V |
| Hb^n^ |  | V |  | V |  |
| Hct^o^ |  |  | V | V |  |
| MCV^p^ |  |  | V | V |  |
| MCHC^q^ |  | V |  | V |  |
| MCH^r^ | V |  |  | V |  |
| **Previous lab test** |  |  |  |  |  |
| AST |  |  | V | V | V |
| ALT | V | V |  | V |  |
| SCr |  |  | V | V |  |
| Bilirubin |  |  |  | V |  |
| RBC |  |  | V | V |  |
| Hb | V |  |  | V |  |
| Hct |  |  |  | V | V |
| MCV |  |  |  | V | V |
| MCHC | V |  |  | V |  |
| MCH |  |  |  | V |  |
| **Slope of lab test** |  |  |  |  |  |
| AST | V |  |  | V |  |
| ALT |  | V |  | V |  |
| SCr | V |  |  | V |  |
| Bilirubin |  | V |  | V |  |
| RBC |  |  |  | V | V |
| Hb |  |  |  | V |  |
| Hct | V | V |  |  | V |
| MCV | V | V |  | V |  |
| MCHC | V |  |  |  |  |
| MCH |  |  |  | V |  |

^a^BMI: Body mass index

^b^HCC: Hepatocellular carcinoma

^c^RCC: Renal cell carcinoma

^d^CKD: Chronic Kidney Disease

^e^PPI: Proton-pump inhibitor

^f^NSAIDs: Non-steroidal anti-inflammatory drugs

^g^AntiDM: Antidiabetic drugs

^h^TSH: Thyroid stimulating hormone

^i^AST: Aspartate aminotransferase

^j^ALT: Alanine transaminase

^k^SCr: Serum creatinine

^l^TG: Triglyceride

^m^RBC: Red blood cell

^n^Hb: Hemoglobin

^o^Hct: Hematocrit

^p^MCV: Mean corpuscular volume

^q^MCHC: Mean corpuscular haemoglobin concentration

^r^MCH: Mean corpuscular haemoglobin
