# Supplementary material for: Web-Based Explainable Machine Learning-Based Drug Surveillance for Predicting Sunitinib- and Sorafenib-Associated Thyroid Dysfunction: Model Development and Validation Study
Source: JMIR Form Res. 2025 Apr 10;9:e67767. doi: 10.2196/67767 (PMC12005597; doi:10.2196/67767)
Supplement: Multimedia Appendix 8 [file formative-v9-e67767-s008.docx]

**Multimedia Appendix 8.1.** Model performance of five-fold cross-validation

|  | **AUROC^i^** | **AUPRC^j^** | **F1 score** | **Recall** | **Precision** | **Accuracy** |
| --- | --- | --- | --- | --- | --- | --- |
| GBDT^a^_RAW | 0.738  [0.687—0.789] | 0.373  [0.260—0.486] | 0.168  [0.000—0.462] | 0.118  [0.000—0.375] | 0.420  [0.000—1.000] | 0.913  [0.885—0.943] |
| GBDT_ROS^b^ | 0.709  [0.661—0.758] | 0.309  [0.173—0.446] | 0.206  [0.000—0.400] | 0.140  [0.000—0.250] | 0.483  [0.000—1.000] | 0.913  [0.902—0.926] |
| GBDT_BSMT^c^ | 0.657  [0.624—0.690] | 0.227  [0.146—0.308] | 0.228  [0.100—0.348] | 0.232  [0.125—0.364] | 0.245  [0.083—0.375] | 0.870  [0.852—0.885] |
| GBDT_STMK^d^ | 0.690  [0.644—0.736] | 0.248  [0.152—0.344] | 0.165  [0.000—0.385] | 0.163  [0.000—0.312] | 0.187  [0.000—0.500] | 0.877  [0.869—0.893] |
| ADA^e^_RAW | 0.725  [0.671—0.780] | 0.283  [0.153—0.413] | 0.143  [0.000—0.381] | 0.093  [0.000—0.250] | 0.327  [0.000—0.800] | 0.897  [0.868—0.926] |
| ADA_ROS | 0.687  [0.642—0.732] | 0.259  [0.140—0.379] | 0.268  [0.095—0.533] | 0.273  [0.143—0.500] | 0.273  [0.071—0.571] | 0.872  [0.843—0.885] |
| ADA_BSMT | 0.719  [0.678—0.761] | 0.267  [0.153—0.381] | 0.264  [0.087—0.457] | 0.338  [0.143—0.500] | 0.222  [0.062—0.421 | 0.841  [0.826—0.844] |
| ADA_STMK | 0.708  [0.646—0.770] | 0.262  [0.150—0.374] | 0.233  [0.174—0.378] | 0.320  [0.182—0.438] | 0.190  [0.133—0.333] | 0.829  [0.803—0.844] |
| LGBM^f^_RAW | 0.706  [0.625—0.787] | 0.226  [0.140—0.312] | 0.130  [0.000—0.211] | 0.099  [0.000—0.143] | 0.258  [0.000—0.667] | 0.893  [0.877—0.926] |
| LGBM_ROS | 0.694  [0.644—0.745] | 0.202  [0.137—0.268] | 0.085  [0.000—0.308] | 0.062  [0.000—0.250] | 0.280  [0.000—1.000] | 0.905  [0.877—0.926] |
| LGBM_BSMT | 0.682  [0.619—0.745] | 0.185  [0.126—0.243] | 0.136  [0.000—0.261] | 0.131  [0.000—0.200] | 0.162  [0.000—0.429] | 0.862  [0.844—0.876] |
| LGBM_STMK | 0.685  [0.613—0.758] | 0.168  [0.115—0.222] | 0.111  [0.000—0.240] | 0.106  [0.000—0.200] | 0.128  [0.000—0.333] | 0.860  [0.844—0.877] |
| RF^g^_RAW | 0.693  [0.626—0.760] | 0.194  [0.120—0.268] | 0.239  [0.130—0.386] | 0.560  [0.273—0.714] | 0.154  [0.086—0.268] | 0.706  [0.672—0.762] |
| RF_ROS | 0.686  [0.609—0.762] | 0.175  [0.113—0.237] | 0.253  [0.133—0.393] | 0.608  [0.273—0.857] | 0.162  [0.088—0.275] | 0.708  [0.664—0.746] |
| RF_BSMT | 0.673  [0.613—0.733] | 0.188  [0.115—0.262] | 0.133  [0.000—0.333] | 0.145  [0.000—0.286] | 0.154  [0.000—0.500] | 0.839  [0.811—0.869] |
| RF_STMK | 0.686  [0.616—0.756] | 0.198  [0.133—0.263] | 0.130  [0.000—0.286] | 0.145  [0.000—0.286] | 0.126  [0.000—0.333] | 0.837  [0.803—0.868] |
| LR^h^_RAW | 0.762  [0.701—0.824] | 0.264  [0.145—0.384] | 0.250  [0.154—0.400] | 0.715  [0.455—0.875] | 0.194  [0.102—0.333] | 0.711  [0.590—0.779] |
| LR_ROS | 0.764  [0.705—0.823] | 0.264  [0.148—0.380] | 0.217  [0.089—0.393] | 0.766  [0.545—0.875] | 0.227  [0.133—0.400] | 0.745  [0.639—0.820] |
| LR_BSMT | 0.725  [0.661—0.790] | 0.235  [0.139—0.330] | 0.237  [0.146—0.333] | 0.456  [0.182—0.625] | 0.171  [0.054—0.345] | 0.755  [0.639—0.795] |
| LR_STMK | 0.733  [0.669—0.797] | 0.252  [0.157—0.347] | 0.220  [0.122—0.314] | 0.537  [0.364—0.625] | 0.197  [0.108—0.345] | 0.769  [0.672—0.810] |

*Values are demonstrated by mean [ minimum – maximum ] of 5-fold cross-validation.

^a^GBDT: Gradient Boosting Decision Tree

^b^ROS: Random oversampling

^c^BSMT: Oversampling with Borderline Synthetic Minority Oversampling Technique

^d^STMK: Synthetic Minority Oversampling Technique-Tomek Links

^e^ADA: Adaptive boosting

^f^LGBM: Light Gradient Boosting Machine

^g^RF: Random forest

^h^LR: Logistic regression

iAUROC: Area under the receiver operating characteristic curve

jAUPRC: Area under the precision-recall curve

**Multimedia Appendix 8.2.** Statistical significance tests of AUPRC

| **Model** | **AUPRC [95% CI]** | **Difference in areas (95% CI)** |
| --- | --- | --- |
| GBDT^a^_RAW | 0.600 [0.350-0.798] | Reference |
| GBDT_ROS^b^ | 0.388 [0.175-0.618] | 0.2237 (0.1093-0.3478) |
| GBDT_BSMT^c^ | 0.300 [0.124-0.549] | 0.3003 (0.1628-0.4801) |
| GBDT_STMK^d^ | 0.346 [0.147-0.582] | 0.2647 (0.1313-0.4029) |
| ADA^e^_RAW | 0.363 [0.159-0.597] | Reference |
| ADA_ROS | 0.316 [0.134-0.564] | 0.0369 (-0.1139-0.1813) |
| ADA_BSMT | 0.383 [0.180-0.624] | 0.03052 (-0.07611-0.1888) |
| ADA_STMK | 0.347 [0.154-0.591] | 0.006941 (-0.1395-0.1080) |
| LGBM^f^_RAW | 0.262 [0.095-0.505] | Reference |
| LGBM_ROS | 0.258 [0.093-0.502] | 0.003967 (-0.01802-0.04029) |
| LGBM_BSMT | 0.304 [0.124-0.549] | 0.04621 (-0.005034-0.1770) |
| LGBM_STMK | 0.207 [0.058-0.441] | 0.06595 (0.01217-0.1982) |
| RF^g^_RAW | 0.342 [0.148-0.583] | Reference |
| RF_ROS | 0.250 [0.083-0.485] | 0.1047 (0.01943-0.2700) |
| RF_BSMT | 0.198 [0.057-0.439] | 0.1511 (0.04367-0.3129) |
| RF_STMK | 0.224 [0.068-0.460] | 0.1301 (0.04086-0.2783) |
| LR^h^_RAW | 0.312 [0.235-0.551] | Reference |
| LR_ROS | 0.329 [0.136-0.566] | 0.01642 (-0.05914-0.05428) |
| LR_BSMT | 0.242 [0.084-0.487] | 0.06772 (0.01022-0.1916) |
| LR_STMK | 0.300 [0.121-0.545] | 0.006236 (-0.09704-0.07534) |

^a^GBDT: Gradient Boosting Decision Tree

^b^ROS: Random oversampling

^c^BSMT: Oversampling with Borderline Synthetic Minority Oversampling Technique

^d^STMK: Synthetic Minority Oversampling Technique-Tomek Links

^e^LGBM: Light Gradient Boosting Machine

^f^ADA: Adaptive boosting

^g^RF: Random forest

^h^LR: Logistic regression
